# Glycans as Targets for Drug Delivery in Cancer

**DOI:** 10.3390/cancers14040911

**Published:** 2022-02-12

**Authors:** Francisca Diniz, Pedro Coelho, Henrique O. Duarte, Bruno Sarmento, Celso A. Reis, Joana Gomes

**Affiliations:** 1 i3S—Instituto de Investigação e Inovação em Saúde, Universidade do Porto, 4200-135 Porto, Portugal; fdiniz@ipatimup.pt (F.D.); pcoelho@i3s.up.pt (P.C.); hduarte@ipatimup.pt (H.O.D.); bruno.sarmento@i3s.up.pt (B.S.); 2 IPATIMUP—Institute of Molecular Pathology and Immunology, University of Porto, 4200-135 Porto, Portugal; 3 ICBAS—Instituto de Ciências Biomédicas Abel Salazar, Universidade do Porto, 4050-313 Porto, Portugal; 4 INEB—Instituto Nacional de Engenharia Biomédica, Universidade do Porto, 4200-135 Porto, Portugal; 5 CESPU—Instituto de Investigação e Formação Avançada em Ciências e Tecnologias da Saúde, 4585-116 Gandra, Portugal; 6 Department of Pathology, Faculty of Medicine, University of Porto, 4200-319 Porto, Portugal

**Keywords:** antibodies, cancer, drug delivery, glycans, glycosylation, nanoparticles

## Abstract

**Simple Summary:**

Alterations in glycosylation are frequently observed in cancer cells. Different strategies have been proposed to increase drug delivery to the tumor site in order to improve the therapeutic efficacy of anti-cancer drugs and avoid collateral cytotoxicity. The exploitation of drug delivery approaches directed to cancer-associated glycans has the potential to pave the way for better and more efficient personalized treatment practices. Such strategies taking advantage of aberrant cell surface glycosylation patterns enhance the targeting efficiency and optimize the delivery of clinically used drugs to cancer cells, with major potential for the clinical applications.

**Abstract:**

Innovative strategies have been proposed to increase drug delivery to the tumor site and avoid cytotoxicity, improving the therapeutic efficacy of well-established anti-cancer drugs. Alterations in normal glycosylation processes are frequently observed in cancer cells and the resulting cell surface aberrant glycans can be used as direct molecular targets for drug delivery. In the present review, we address the development of strategies, such as monoclonal antibodies, antibody–drug conjugates and nanoparticles that specific and selectively target cancer-associated glycans in tumor cells. The use of nanoparticles for drug delivery encompasses novel applications in cancer therapy, including vaccines encapsulated in synthetic nanoparticles and specific nanoparticles that target glycoproteins or glycan-binding proteins. Here, we highlight their potential to enhance targeting approaches and to optimize the delivery of clinically approved drugs to the tumor microenvironment, paving the way for improved personalized treatment approaches with major potential importance for the pharmaceutical and clinical sectors.

## 1. Introduction

Cancer remains one of the deadliest diseases and a major burden worldwide. Over the last decades, early detection and effective treatment of cancer have been the main focus in the field. Indeed, major advances in the fields of molecular biology and the cellular basis of cancer development and progression have been made and contributed to a more comprehensive understanding of the disease [1].

Chemotherapy comprises powerful drugs against cancer that target key biological mechanisms leading to the killing of highly proliferative cells in body. However, the use and application of these drugs in cancer treatment have significant limitations, mostly related to the dose-limiting toxicity, poor specificity towards tumor cells and the frequent emergence of drug resistance [2,3].

Targeting cancer cells is crucial to improve the efficiency of specific drugs, avoiding drug-induced toxicity in non-neoplastic tissues, but also to aid in the detection of cancer cells and, consequently, improving diagnostics. For this reason, the development of novel and more efficient strategies to specifically target malignant cells remains of major interest [4,5].

The current concept at the basis of precision oncology is that tumor-specific molecular abnormalities can be targeted with accurate, effective, and potentially less-toxic therapies. The first gateways for genomic accuracy in oncology were opened upon large preclinical work and primary discoveries of somatic, single-gene genomic abnormalities underlying oncogenic transformation that could be pharmacologically targeted. The resulting aberrantly expressed proteins can be selectively targeted by strategies using specific compounds and drugs. Recently, the use of aberrant glycans overexpressed by cancer cells have emerged as potential molecular candidates for the improvement of cancer targeted therapies [6,7].

## 2. Glycosylation in Cancer

**Figure 1:**
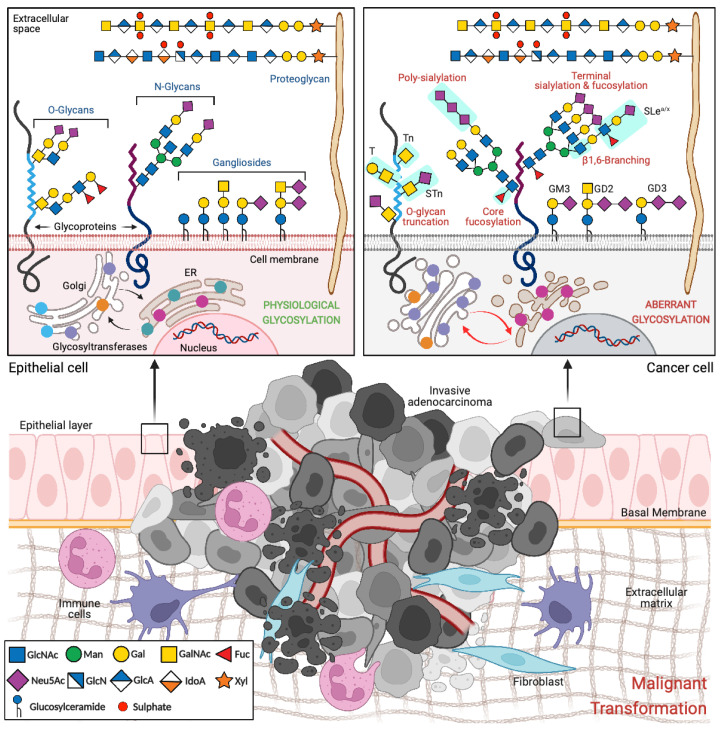
Glycosylation alterations during malignant transformation. Abbreviations: ER—endoplasmic reticulum; Fuc—fucose; Gal—galactose; GlcN—glucosamine; GlcA—glucuronic acid; GlcNAc—N-acetylglucosamine; GalNAc—N-acetylgalactosamine; IdoA—iduronic acid; Man—mannose; Neu5Ac—N-acetylneuraminic acid; STn—sialyl Tn; SLe^a/x^—sialyl Lewis a/x; T—Thomsen–Friedenreich antigen; Xyl—xylose.

Glycosylation is a highly regulated enzymatic process comprising the addition of an initial monosaccharide to a non-glycosyl aglycone, usually a protein or a lipid. The initial sugar is further elongated with additional monosaccharides, which are linked covalently through glycosidic bonds, forming oligosaccharides or polysaccharides. Glycosylation occurs in the endoplasmic reticulum (ER) and Golgi apparatus, where the highly orchestrated and organized activity of both glycosyltransferases and glycosidases produces a vast repertoire of structurally complex and functionally diverse carbohydrate structure, or glycans [8] (Figure 1).

In protein glycosylation, glycans are typically attached via glycosidic linkages to the nitrogen atom on the side chain of asparagine (N-glycans) [9] or the oxygen atom on the side chain of threonine or serine (O-glycans) [10]. Other important type of glycoconjugates are glycosphingolipids (GSLs), cell-surface glycolipids formed by a hydrophobic ceramide core and a hydrophilic residue [11]. On the other hand, proteoglycans are glycoconjugates with one or more glycosaminoglycan (GAG) chains, such as chondroitin sulfate, heparan sulfate and keratan sulfate, attached to a protein backbone [12].

Glycans are produced in a non-templated way and their synthesis is controlled by substrate availability, gene transcription levels, tissue-specific expression patterns of glycosyltransferases and glycosidases, enzyme location within the compartments of the secretory pathway, chaperone activity, donor substrate availability, and modifications in the tertiary conformation of acceptor peptides [13,14].

Cell surface glycoconjugates form the glycocalyx, a dense gel-like protective barrier that shields the plasma membrane from physical stress while also shaping various aspects of cell surface dynamics. Furthermore, cell surface glycoconjugates are key regulators of various molecular and cellular processes, including intracellular trafficking, cell–cell interactions, cell–matrix adhesion, as well as molecular signaling, immune regulation, host-pathogen recognition and malignant transformation [13] (Figure 1).

In cancer, aberrant expression and mislocalization of glycosyltransferases and glycosidases, as well as imbalances affecting the availability of sugar nucleotide donors, lead to the emergence of aberrant glycosylation patterns (Figure 1). These cancer-derived glycan structures are key players in tumor biology, as they actively support to the various neoplastic characteristics of malignant cells [15]. In fact, it has been extensively demonstrated that cancer-associated glycans mediate the crosstalk between cancer cells and their surrounding microenvironment, contributing for the acquisition of cancer hallmarks, such as sustained proliferative signaling, resistance to cell death, immune system evasion, angiogenesis, invasion and metastization [13] (Figure 1). Receptor tyrosine kinases (RTKs) and their downstream signaling cascades actively drive neoplastic transformation. Moreover, several studies have demonstrated that the altered glycosylation of different RTKs (such as EGFR, ErbB2, Met, and RON) leads to their hyperactivation, which, in turn, promotes RTK-dependent malignant growth and phenotypically aggressive tumors [16,17,18,19,20].

### 2.1. Glycans with Specific Expression in Cancer

Aberrant glycosylation in cancer leads to the biosynthesis of tumor-associated glycans that underpin several oncogenic features leading to tumor onset and progression. Altered glycosylation encompasses the synthesis of prematurely truncated O-glycans, such as the single monosaccharide N-acetylgalactosamine (GalNAc; Tn antigen), sialyl Tn (STn) and the Thomsen–Friedenreich (T) antigen, as well as an increase in N-glycan β1,6-branching, terminal sialylation, including the biosynthesis of polysialic acid-containing structures, and both core and antennae-linked fucosylation. These alterations support the enrichment of cell surface glycoconjugates with sialofucosylated Lewis antigens (sialyl Lewis x (SLe^x^) and sialyl Lewis a (SLe^a^)) [13] (Figure 1). These tumor-associated glycan structures can be present on the surface of cancer cells, but they can be also secreted or shed into the circulation and, thus, serve as potential biomarkers of disease (such as SLe^a^ serologically detected by CA19-9, STn detected by CA72-4, MUC16 detected by CA125 and MUC1 detected by CA15-3) [13]. Due to their unique characteristics and highly restricted expression in cancer, these tumor-associated glycans are emerging as promising targets in the context of targeted therapies.

#### 2.1.1. Truncated O-Glycans

O-glycosylation produces mature, elongated, and branched O-glycan chains, often terminally modified with sialic acids or N-acetylneuraminic acid (Neu5Ac) through the action of sialyltransferases. A set of 20 GalNAc-transferases (encoded by *GALNT*s) initiates the biosynthesis of O-GalNAc glycan chains [21]. This family of enzymes transfers GalNAc to Ser/Thr residues of proteins, forming the Tn antigen (GalNAcα1-O-Ser/Thr). Subsequently, the Tn antigen is modified by different glycosyltransferases, forming complex and mature O-glycans. Glycosyltransferase core 1 GalNAc β1,3-galactosyltransferase 1 (C1GalT1) is responsible for adding galactose (Gal) to the Tn antigen, forming the core 1 O-glycan structure or the T antigen (Galβ1-3GalNAcα1-O-Ser/Thr). Core 1 β3-Gal-T specific molecular chaperone (COSMC), localized in the ER, is necessary for the activation of C1GalT1 in the Golgi apparatus by preventing its misfolding, aggregation and proteasomal degradation [22]. Core 1 structures can be further modified by core 2 β1,6-N-acetylglucosaminyltransferase (C2GnT), which adds N-acetylglucosamine (GlcNAc) to the T antigen, forming core 2 O-glycans. These structures can then be subsequently modified by different glycosyltransferases, including sialyl- and fucosyltransferases, forming increasingly complex structures [10].

In cancer, alterations affecting the glycosylation machinery trigger the expression of immature and truncated O-glycans, such as Tn antigen, and its sialylated form, STn (NeuAcα2-6-GalNAcα1-O-Ser/Thr) [13]. In non-transformed cells, early-acting enzymes responsible for core O-glycan biosynthesis, such as C1GalT1 and C2GnT, are localized in cis- and medial-Golgi, and late-acting enzymes, such as sialyltransferases, are localized in the trans-Golgi. In the cancer setting, however, the overexpression of sialyltransferases leads to their aberrant distribution across all of the Golgi cisternae, where they will compete with early acting-enzymes and contribute to the emergence of immature forms of O-glycan chains. One example is the overexpression of the α-GalNAc α2,6-sialyltransferase I (ST6GalNAc1) that leads to the premature addition of sialic acid to form the STn antigen [23,24].

While STn expression is neglectable in normal tissues, high levels of this short O-glycan antigen have been widely reported across different epithelial-derived tumors, such as gastric, pancreatic, colorectal, and breast, and are associated with poor patient survival [25]. The mechanisms involved in STn biosynthesis include loss of C1GalT1 expression, mutations and hypermethylation of COSMC, and overexpression of ST6GalNAc1 [24,26]. STn overexpression in gastrointestinal cancer cells leads to a more aggressive phenotype, supporting the epithelial-to-mesenchymal transition (EMT) process and reducing cell–cell adhesion capacity through the loss of E-cadherin expression [27,28,29]. Furthermore, STn-overexpressing cells are more capable of degrading and invading the surrounding extracellular matrix (ECM), being associated with poor clinical outcome and chemotherapy resistance [27,28,29,30].

In cancer, several glycoproteins are overexpressed and aberrantly glycosylated, carrying tumor-associated glycans underpinning tumor growth and metastization [15]. CD44, a transmembrane receptor for hyaluronic acid (HA) and other ECM molecules, is a well-known glycoprotein, often overexpressed in several cancers and reported as a major carrier of truncated O-glycans in gastric cancer cells. The presence of these truncated O-glycans impact CD44 binding to HA, while also promoting hyperactivation of RON receptor [31]. Another prominent glycoprotein in the context of cancer is MUC1, a highly glycosylated transmembrane mucin, that has also been reported to be a carrier of STn in multiple cancer types, and whose overexpression has been linked to tumor growth and metastization [32].

#### 2.1.2. Sialylated Glycans

One of the main terminal modifications of glycans is the addition of a negatively charged acid (Neu5Ac) to glycoproteins and GSLs, contributing to their biophysical and biological functions. Sialylation of glycoproteins plays key roles in the blood and lymphatic vasculature, being essential for cell–cell and glycoprotein–protein interactions [33,34,35]. The SLe^x^ and SLe^a^ isomers mediate, under physiological conditions, the binding of circulating leukocytes to cell surface selectins expressed on activated endothelial cells during immune responses [36]. A frequent alteration that occurs in several cancers is the increased expression of SLe^x^ and SLe^a^. As such, tumor cells bind to vascular endothelial selectins, enhancing their migration and extravasation [37,38]. ST3Gal3, ST3Gal4 and ST3Gal6 are upregulated in different cancer types and drive the expression of SLe^x^. In gastric cancer, high expression of ST3Gal4 is associated with an increased capacity of tumor cells to invade and metastasize. In addition, SLe^x^ expression in tumor cells has been reported to promote proliferation, motility, angiogenesis, while protecting tumor cells from apoptosis [20].

The glycoprotein carcinoembryonic antigen (CEA) is an oncofetal antigen expressed in the developing fetus and in epithelial malignancies, but only in neglectable amounts in normal adult human tissues. In gastric carcinoma, CEA was described as the main carrier of SLe^x^, and this glycoform was associated with a more aggressive phenotype [39].

Apart from Neu5Ac, others non-human members of sialic acid family, named N-glycolylneuraminic acid (Neu5Gc) and 2-keto-3-deoxy-D-glycero-D-galacto-nononic acid (KDN), although deficient or less abundant than Neu5Ac in humans, have also been detected with elevated levels in human cancers [40,41,42].

### 2.2. Glycan-Binding Proteins

Immune cells express glycan-binding protein (GBP) receptors, such as galectins, C-type lectin receptors (e.g., selectins) and sialic acid-binding immunoglobulin-like lectins (Siglecs) that, upon the recognition of specific glycan ligands, activate tolerogenic or immunogenic signaling pathways [43,44]. The Siglec family consists of 15 transmembrane proteins expressed by most immune cells, and can be classified into two groups: the CD33-related Siglecs with high sequence identity (Siglec-3, -5, -6, -7, -8, -9, -10, -11) and the structurally conserved Siglecs (Siglec-1, -2, -4 and -15) [45]. Recognition of tumor-associated glycans by GBPs expressed on immune cells leads to production of anti-inflammatory cytokines, inhibits natural killer (NK) cell-mediated cytotoxicity, and activates immunosuppressive regulatory T cells [45]. For example, Siglecs are differentially expressed by immune cells and are capable of recognizing sialic acid motifs in a linkage-specific pattern (α2,3, α2,6 or α2,8) [43,46]. Upon binding to sialic acids, Siglecs’ intracellular immunoreceptor tyrosine-based inhibition motifs induce strong inhibitory signals that suppress the immune response [46,47].

## 3. Specific Targeting of Cancer Cells

**Figure 2:**
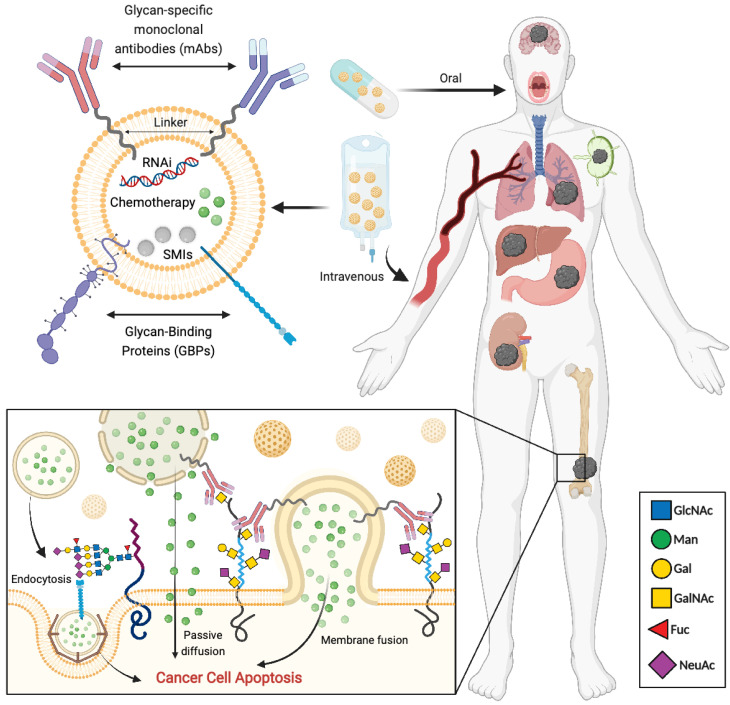
Strategies based on targeting cancer-associated glycans for selective and specific drug delivery systems.

The timely and accurate diagnosis and treatment of cancer requires the unequivocal in vivo detection and targeting of neoplastic cells. Indeed, the process of malignant transformation of healthy cells is underpinned by a plethora of molecular aberrations occurring at the genomic, epigenomic and post-translational levels. Such oncogenic events, many of which are therapeutically actionable, trigger the emergence of an array of both cell surface and intracellular tumor-specific neoantigens, whose expression is undetectable or neglectable in non-transformed cells. The highly restricted expression of such neoantigens within the cancer cell population makes them suitable molecular targets for antibody-based recognition, as well as appealing candidates for the development of both targeted therapeutic strategies and imaging systems for disease diagnosis. Since most therapeutic strategies for targeted drug delivery rely on the recognition of cell surface-residing molecules, the ideal target antigen should be broadly expressed at the membrane of tumor cells, while absent from the cell surface of healthy cells. Thus, the expression of aberrantly glycosylated forms of cell surface glycoconjugates in cancer cells makes these molecular entities appealing targets for highly selective and specific drug delivery systems (reviewed in [6,13]) (Figure 2).

### 3.1. Monoclonal Antibodies

Monoclonal antibodies (mAbs) targeting antigens that are either unique to (neoantigens) or overexpressed by malignant tissues trigger cancer cell death through a variety of effector mechanisms [48]. These include the blocking of receptor-induced proliferative signaling, the activation of anti-tumor immune responses, including antibody-dependent cellular cytotoxicity (ADCC), complement-dependent cytotoxicity (CDC) and antibody-dependent cellular phagocytosis (ADCP). Antibody-triggered immune-mediated responses require the engagement of the fragment crystallizable (Fc) region of the therapeutic mAb with multiple Fc receptors (FcRs) expressed at the surface of distinct immune cell populations, including NK cells, neutrophils, monocytes, dendritic cells and eosinophils [49]. Such interaction triggers the activation of a repertoire of specialized functions aiming at eliminating the cell to which the mAb is bound. Importantly, the immunoglobulin subclass further determines the capacity of a given therapeutic mAb to elicit immune-mediated effector responses. For instance, mAbs belonging to the IgG1 and IgG3 subclasses are able to induce the activation of ADCC and CDC, while IgG2 and IgG4 are not [50]. Several clinically approved mAbs target key immune checkpoints, such as the ones mediated by CTLA-4 and PD-1/PD-L1, and aim at blocking inhibitory self-tolerance signals. Such immunotherapies counteract the establishment of an immunosuppressive microenvironment and, thus, the escape of tumor cells to immune surveillance [51].

The advent of the hybridoma technology nearly 50 years ago allowed the generation of a virtually unlimited catalog of highly specific mAbs for the reliable detection of protein-, lipid- and glycan-based tumor-specific antigens. In the hybridoma system, during the immunization of a host organism, naive antibody-secreting B-cells are challenged with the purified target antigen [52,53]. Subsequently, activated cell clones are fused with myeloma cells for immortalization, generating a hybrid, clonal and antibody-producing cell line, or hybridoma. Additional phage display technology has also been applied for the production of antibodies [54,55]. The resulting mAbs can then undergo chimerization or, more recently, full humanization to reduce their immunogenicity, and are subsequently used as targeted therapeutic agents [56]. In the context of biomarker-guided precision oncology, the detection of the target antigen in individual tumor specimens is warranted to determine patient eligibility for mAb-based therapeutic regimens. Such is the case of clinically approved therapeutic mAbs trastuzumab and pembrolizumab/nivolumab, used in the treatment of multiple ErbB2- and PD-L1-expressing solid tumors, respectively [57].

Over the past three decades, more than 20 therapeutic mAbs have been granted Food and Drug Administration (FDA) approval for the treatment of both solid and circulating tumors, most of which belong to the IgG1 subclass [49]. Most mAbs approved in the clinical setting target oncogenic RTKs and their cognate ligands, whose overexpression actively upregulates transcriptomic networks sustaining cancer cell proliferative signaling and angiogenic growth. Amongst the most prominent molecular targets are: two members of the epidermal growth factor receptor family (EGFR and ErbB2); the vascular endothelial growth factor receptors and ligands (VEGFR2 and VEGF); platelet-derived growth factor receptors (PDGFRα); and key immune checkpoint regulators (PD-1/PD-L1, CTLA-4). Some mAbs directed at cancer-derived glycopeptides and other glycan-based antigens have shown promising selectivity towards cancer cells and potent cytotoxicity. These include mAbs generated against Tn/STn-modified MUC1 and MUC16 glycopeptides, and the clinically approved dinutuximab targeting the GD2 GSL, approved in the clinical setting as a second-line therapeutic agent for the treatment of high-risk neuroblastoma [58,59,60,61].

Although the clinical implementation of such compounds has proven successful in the treatment of cancer, both innate and acquired molecular resistance continue to pose a significant challenge to the efficacy of therapeutic mAbs. These include the downregulation of the cell surface molecule targeted by the mAb [62], the activation of bypass signaling circuits that circumvent mAb-mediated receptor blockade [63], and the acquisition of mutations in the target molecule that either prevent mAb binding or significantly reduce target affinity [64,65]. Furthermore, due to the poor vascular irrigation and impaired lymphatic drainage of most solid tumors, efficient extravasation, tissue penetration and homogeneous distribution of systemically administered mAbs at the target tumor site have proven challenging to achieve [66]. Furthermore, glycans have a major impact on target epitope recognition for cancer diagnosis and therapeutic stratification [6,67,68]. Indeed, glycan-mediated epitope masking hampers the in situ detection of therapeutic targets such as the PD-L1 immune checkpoint [68]. Enzymatic glycan removal improves PD-L1 detection in cancer patient tissues and predicts the clinical benefit from immunotherapeutic regimens [69].

The stability, immunogenicity and biological activity of therapeutic mAbs can be further optimized through glycoengineering, i.e., by rigorously controlling the glycosylation status of conserved sites at the Fc region [70]. Indeed, subtle changes in the cell culture and downstream processing procedures significantly influence the glycosylation pattern of produced mAbs, which, in turn, crucially impacts their structural and functional properties. MAbs of the IgG class harbor a conserved site for N-linked glycosylation at the Asn297. For instance, the removal of core fucose from this glycosylation site, or fucosylation, has been shown to increase the binding affinity of IgG1 mAbs to their cognate FcγRIIIa receptor through reduction of steric hindrance [71,72]. The decoration of complex N-glycan structures with terminal Neu5Ac motifs, on the other hand, inhibits the binding affinity of mAbs to the FcγRIIIa receptor and, consequently, dampens the activation of ADCC responses [73]. Terminal galactosylation of glycan chains decorating Asn297 has been reported to induce CDC-based immune responses [74]. Additionally, specific glycosylation profiles of therapeutic mAbs crucially govern their pharmacokinetic properties. For instance, the modification of Asn297 with oligomannosidic, or high-mannose, N-glycan species confers therapeutic mAbs faster clearance rates [75].

With the exceptions of mAbs targeting CD20, ErbB2 and EGFR, most therapeutic mAbs approved in the clinical setting bear modest cytotoxic activity on their own. Regardless, given their high specificity to tumor-derived antigens, mAbs represent extremely versatile platforms for the targeted delivery of cytotoxic drugs to the tumor site, such as conventional chemotherapeutic agents (antibody-drug conjugates (ADCs)), while reducing the occurrence of off-target toxicity. More recently, therapeutic antibodies have been engineered to bind to more than just two structurally identical epitopes (multi-valency), as well as to structurally distinct epitopes (multi-specificity) [76]. Such is the example of bi-specific T cell Engagers (BiTEs), which aim at stimulating anti-tumor immune-mediated synapses by simultaneously targeting a tumor antigen and the activating T cell receptor CD3 [77]. Other bi-specific mAb formulations aim at effectively neutralizing RTK-induced oncogenic signaling, by blocking cell surface activation of multiple target receptors [78].

### 3.2. Antibody-Drug Conjugates

The specificity of mAbs towards their cognate antigen can be advantageous in the targeted delivery of non-specific cytotoxic agents to cancer cells, whilst avoiding healthy tissues. Such ADC formulations currently represent the fastest growing drug class in oncology. These therapeutic formulations are composed of three main elements: a cytotoxic drug (also known as cytotoxic payload), a mAb and a flexible linker [79]. The mechanistic principle underlying this strategy is based on the highly specific recognition of a cellular surface antigen, whose expression is highly restricted to the cancer cell population, by the mAb moiety, and the subsequent delivery of the cytotoxic payload to the tumor tissues. Such systems can, therefore, improve the efficacy of chemotherapy and reduce systemic exposure to the drug’s toxicity and side effects [80]. ADCs exert cytotoxicity through two distinct mechanisms. The primary mechanism consists in the cellular internalization of the ADC, followed by the intracellular release of the payload after linker cleavage. The secondary mechanism takes place when the payload or payload-linker permeates adjacent cells, including those with absent expression of the target antigen [79,81].

Until now, nine ADCs have been approved by FDA (Table 1). However, only five have so far received regulatory approval by both the FDA and the European Medicines Agency (EMA) for the treatment of cancer: brentuximab-vedotin, trastuzumab-emtansine (TDM-1), inotuzumab-ozogamicin, gemtuzumab-ozogamicin, and polatuzumab-vedotin. Moreover, dozens of other ADCs are currently under preclinical and clinical stages of development [79,80]. ADCs are thus becoming an important and viable strategy for targeting cancer cells and deliver highly cytotoxic drugs in a highly targeted manner, while protecting the integrity of non-malignant tissues.

The flexible linker of ADCs plays two important roles: ensuring that the cytotoxic payload is firmly connected to the antibody, and allowing its efficient release at the target tumor site [79,80]. It is essential that linkers sustain the stability of the ADC during blood circulation to guarantee its integrity until the target tumor site is reached. Linkers can be classified into two groups: cleavable and non-cleavable linkers. The cleavage of some linkers is dependent on physiological conditions, such as pH of the recipient cell. On the other hand, non-cleavable linkers confer the ADC improved stability while in circulation, increasing the ADC systemic half-life and decreasing off-target toxicity [80]. Of the nine currently FDA-approved ADCs (Table 1), two contain non-cleavable linkers: TDM-1 [82] (indicated for ErbB2-positive metastatic breast cancer) and belantamab-mafodotin [83] (approved for relapsed or refractory multiple myeloma).

Early developed ADCs were design to carry traditional chemotherapeutic drugs (methotrexate, doxorubicin and vinca alkaloids). However, most of these formulations had compromised efficiency which required extremely high dosing [79,80]. Currently, the most widely used payloads, such as auristatins, calicheamicins, maytansinoids and camptothecin analogues, depict higher potency at sub-nanomolar concentrations. The most commonly used payloads target either DNA replication or tubulins. Considering the nine FDA-approved ADCs, only two of them do not target DNA or tubulins: trastuzumab-deruxtecan [84] (approved for metastatic ErbB2-positive breast cancer) and sacituzumab govitecan [85] (indicated for triple-negative breast cancer). The payload target of these two ADCs is topoisomerase I (TOPO1). The strategy of TOPO1 inhibitors, in patients with breast cancer, is being used in advanced stages, contrarily to microtubule-targeting agents which are more broadly used in early lines of treatment. This approach makes the treated tumor more susceptible to the cytotoxicity induced by TOPO1 inhibitors and avoids the emergence of therapeutic resistance [79].

The efficient and successful delivery of ADCs to cancer cells relies on the molecular target. Ideally, the selected target antigen should be widely expressed on the cancer cell surface to enable binding of the circulating ADC and absent or poorly expressed in healthy tissues to minimize off-target toxicity. Nevertheless, the efficacy of ADCs is also dependent of their specific binding affinity or internalization rate [79,80]. The molecular targets of four out of nine approved ADCs are homogenously expressed within the target cancer tissues: inotuzumab ozogamicin (indicated as B cell precursor in ALL and targeting CD22) [86], gemtuzumab ozogamicin (approved for CD33-positive AML and targeting CD33) [87], brentuximab vedotin (indicated in Hodgkin’s lymphoma, ALCL, PTCL and MF and targeting CD30) [88], and polatuzumab vedotin (indicated in DLBCL and targeting CD79) [89]. ErbB2 constitutes a cell surface RTK widely expressed in epithelial-derived tumors. Thus, the use of FDA-approved ADCs for the treatment of ErbB2-addicted solid tumors, which include TDM-1 [82] and trastuzumab-deruxtecan [84], is solely indicated in tumors with confirmed ErbB2 expression.

Most malignant tumors are able to escape immune surveillance and immune-mediated destruction. The cell surface presentation of Neu5Ac by tumor cells constitutes one of the main mechanisms underlying the capacity of tumor cells to evade immune-mediated responses. Thus, strategies targeting tumor-associated sialosides to potentiate anti-cancer immune response constitute promising therapeutic avenues. In line with this, Xiao et al. developed a trastuzumab-sialidase conjugate that selectively removes Neu5Ac moieties from ErbB2-expressing tumor cells, as well as ErbB2-negative neighboring cells, immunoediting their glycocalyx and increasing tumor cell vulnerability to ADCC [90].

### 3.3. Nanoparticles: Application of Nanotherapeutics in Cancer Therapy

In recent years, nanoparticles (NPs) have been extensively explored in biomedical research. Nanomedicines have several advantages over conventional cancer therapies. Firstly, they offer greater efficiency in early detection, diagnosis, imaging, and treatment of cancers [91]. Secondly, nanoscale engineering allows the production of multifunctional and “smart” NPs with extraordinary capacities, such as the ability to cross biological barriers, to target specific cells, and to control the drug release [5]. NPs have been widely used in the cancer field due to their high sensitivity for diagnostic-based imaging but also due to their use as alternative tools for targeted drug delivery with reduced systemic toxicity. The progress and improvements of nanosystems make them promising tools in clinical oncology [92].

The most prominent feature of therapeutic NPs is their capacity to efficiently encapsulate drugs with poor solubility, which they then efficiently deliver to the target tissue, while minimizing off-target toxicity [92] (Figure 2). Indeed, specific characteristics of NPs determine nanocarrier drug delivery efficiency and biodistribution in the body [5]. The most efficient way for a NP to systematically deliver a drug to the target tumor site is by remaining in circulation for a prolonged period. To do so, the nanodrug delivery efficiency can be controlled by modulating NP size and surface physicochemical characteristics [5].

Despite its known toxicity to non-cancer tissues, chemotherapy remains the most widely used therapeutic strategy in the cancer clinical setting. Doxorubicin, 5-fluoroucill and paclitaxel are known as potent anti-cancer drugs for the treatment of multiple cancers. Although these drugs efficiently impair cancer cell proliferation and growth, their systemic side effects and toxicity are also observed in healthy tissues, especially those characterized by significant cellular proliferation and turnover. For these reasons, several potent anti-tumor agents have not progressed into further clinical development [92]. In this context, NPs may represent a valuable alternative to reduce a drug’s off-target adverse effects, by restricting the release of the chemotherapeutic agents to the target tumor site. Nevertheless, there are several challenges related to the use of nanotherapeutic products, such as unintended NP accumulation, as well as pharmacokinetic, physicochemical and safety profiles [93,94,95].

#### Decorating Nanoparticles with Monoclonal Antibodies for Selective and Efficient Cancer Cell Targeting

The type of target tissue is determinant when designing nanodrugs. Nanocarriers can deliver drugs to tumors by two different mechanisms: passive and active targeting.

Passive targeting is accomplished by delivering NPs to defined organs via two different mechanisms, such as the reticuloendothelial system, or the enhanced permeability and retention (EPR) system [93,96]. However, these strategies have some limitations inherent to NP physicochemical properties and the pathophysiological features of cancer tissues. Firstly, the size and surface properties of NPs need to be highly controlled to avoid both renal clearance and uptake by the mononuclear phagocyte system, both of which reduce their circulation half-life. Besides, strict control over the NP size is also important to avoid NP accumulation by the EPR effect at the tumor site, through both convection and diffusion processes. This is expected to affect NPs in the 100–400 nm diameter range [93,95,96]. Furthermore, several chemotherapeutic drugs may induce multiple-drug resistance, which makes anti-cancer therapy become inefficient due to the resistance of cancer cells. This is a consequence of the ineffective drug delivery mechanism that lacks controlled diffusion into the tumor, accumulating NPs improperly in tissues and preventing their prolonged circulation in the bloodstream [5,93]. The passive strategy is further limited in certain hypovascular tumors, which do not exhibit the retention effect and are supplied by vessels depicting heterogeneous permeability and distribution. Besides, the drug delivery process by passive targeting lacks control concerning the random nature of targeting and the inefficient drug diffusion into a tumor. Thus, NPs based on active targeting can help overcome these limitations [93].

Active targeting is based on the conjugation of NPs with molecules that specifically bind to a receptor whose expression is restricted to the target cell population, while depicting neglectable expression in normal cells [5]. The interaction between a ligand/mAb and its cognate receptor may induce NP internalization via receptor-mediated endocytosis and consequent intracellular drug release. Hence, non-specific interactions among NPs and normal cells will be minimized, resulting in decreased systemic side effects and increased cytotoxicity to tumor cells when applied to anti-cancer drug delivery [5,95,96]. Obviously, the success of ligand–receptor interaction depends on the amount of ligand/mAb at the NP surface, ligand orientation, and target receptor expression in the target cells. In this context, increasing the number of binding sites or generating novel binding sites are considered promising strategies to overcome the current limitations of active targeting [5,95,96]. Multiple ligands have been applied to functionalize NPs in the active targeting setting. Still, mAbs, mainly from IgG subtypes, remain the most widely used molecules to achieve active tumor targeting [93,97].

In the early stages of development, full antibodies were used as targeting ligands. Nevertheless, multiple challenges, including immunogenicity, poor stability, fast degradation and lower efficacy further encouraged the use of antibody fragments, which allow for higher loading capacities due to reduction in crowding, superior orientation of targeting ligands and reduced variability in NP diameter constant [93,97]. A NP functionalized with an engineered human Fab targeting the alternatively-spliced CD44v6 was developed, owing to its frequent overexpression in cancer tissues. Their nanodelivery system is characterized by specificity, optimal ligand orientation and low toxicity. Finally, they produced a NP capable of strongly binding to a CD44v6-derived peptide and, more crucially, to cells with endogenous CD44v6 expression, as opposed to CD44v6-negative cells [98,99].

## 4. Sweetening Precision Oncology with Glycan-Directed Nanoparticles

As previously mentioned, aberrant protein glycosylation constitutes an incontrovertible hallmark of the malignant transformation of human cells and tissues [6,13]. Moreover, the restricted expression of glycan-based cell surface neoantigens to cancer cells makes these molecular entities promising targets for selective anti-cancer drug delivery. Over the last decade, several efforts have been made to integrate nanotechnology and carbohydrate field for the targeted and controlled delivery of cancer therapeutic agents. Several studies have demonstrated the importance of using glycosylated NPs for therapeutic purposes (reviewed in [95,100]).

### 4.1. Vaccines Encapsulated in Synthetic Glycan-Targeting Nanoparticles with Glycan Targeting

Over the last decades, the development of vaccines has led to a significant expansion of the human life expectancy, with a positive impact on long-term protective immunity of the population [101]. The mechanism underlying therapeutic cancer vaccines relies on the modulation of the host’s immune response against malignancies. Cancer cells are known to express a series of tumor-associated antigens that suppress the cytotoxic activity of T cell responses. Such is the case of STn- and Tn-decorated MUC1, which is overexpressed in several malignant tumors [95].

NPs can exert optimal actions both as delivery systems and as adjuvants, with the ability to extensively enhance immune responses while ensuring minimal toxicity [102]. A study conducted by Liu et al. [103] produced an anti-tumor vaccine in which a MUC1 glycopeptide was used as the target tumor-associated immunogen, the α-galactosylceramide (α-GalCer) acted as an immune adjuvant, and gold-based NPs (AuNPs) as the multivalent carriers. The developed vaccine triggered a significant and durable antibody response, through specific binding of immunized serum to MUC1-expressing breast cancer cells. In another study, Cai et al. [104] developed a vaccine in which MUC1 glycopeptides were covalently linked to CD4 T-cell peptide epitopes and polyethylene glycol (PEG) combined with AuNPs. This formulation was able to promote antigen presentation by antigen presenting cells, modulating T-cell activation and MHC II-restricted T- and B-cell cooperation. Finally, Mocan et al. [105] demonstrated that AuNPs functionalized with a MUC1 protein fragment generated efficient anti-cancer responses via macrophage activation and polarization towards an inflammatory phenotype (M1). A polymerizable version of the Tn antigen was conjugated to AuNPs and was able to induce the strong and durable production of anti-Tn antibodies, demonstrating that the efficiency of glycan-directed vaccines is comparable to that of formulations targeting entirely peptidic antigens [106].

More recently, a novel antigen delivery system using MUC4, a glycoprotein highly overexpressed in pancreatic tumors, was decorated with the T antigen at different sites. Their synthetic vaccine targets antigen-presenting cells (APC) expressing dectin-1, a C-type lectin known to bind β1,3-glucans. The obtained results showed that these particles induce strong in vivo immune responses, such as production of antibodies and the activation and proliferation of antigen-recognizing T-cells [107].

Some studies also focus on the new strategy based on the selective targets of the mannose receptor expressed in APC, such as dendritic cells and macrophages. It demonstrated a simultaneous enhancement of antigen-specific CD4+ and CD8+ T-cell responses by mannan-decorated poly(lactic-co-glycolic-acid) (PLGA) NPs with ovalbumin encapsulated, in comparison with non-decorated NPs [108]. In addition, drug-free mannosylated liposome NPs were also used to inhibit tumor growth by promoting the polarization of tumor-associated macrophages. It was shown that the induction of the M1 phenotype is associated to the increase of antitumor immune efficacy of immunomodulators [109].

### 4.2. Nanoparticle Strategies Using Glycoproteins

Several reports demonstrated the use of aptamers to target carbohydrate moieties, such as Neu5Ac, instead of mAbs [91,110,111,112,113]. Aptamers are synthetic oligonucleotides or peptide molecules with a small size, lower production cost, high specificity, and reduced immunogenicity [114]. A recent study [115] has developed an anti-MUC1 aptamer conjugated to a chitosan-based NP to co-deliver insulin-like growth factor receptor 1 (IGF-1R) siRNA and the docetaxel chemotherapeutic. This nanoformulation was conjugated with an anti-MUC1 aptamer and was shown to efficiently bind to MUC1-overexpressing metastatic breast cancer cells. Moreover, the developed NP was shown to be efficiently internalized by malignant cells and produce the desired downregulation of IGF-1R expression. Sayari et al. [111] have developed a similar nanocarrier system for the delivery of SN38, the active metabolite of irinotecan, to colon cancer cells. The poor solubility and high toxicity of SN38 have so far precluded its clinical use. However, the encapsulation and specific delivery of this compound to cancer cells may help overcome both these limitations. A MUC1-directed aptamer has been further conjugated with a mesoporous silica NP. The NP was loaded with epirubicin, a cytotoxic drug commonly used to treat breast cancer in the adjuvant setting, and depicted increased uptake and cytotoxicity in breast cancer cells [112]. Perepelyuk et al. evaluated the therapeutic efficacy of MUC1-aptamer functionalized hybrid NPs in lung tumor-bearing mice. This hybrid nanosystem was loaded miRNA-29b, a microRNA targeting DNA methyltransferases, and was shown to increase its specific delivery to the target cells and tissues [113]. Lastly, Yu et al. [110] also reported the potential of a MUC1 protein aptamer when as a targeting agent. Paclitaxel was loaded in aptamer-conjugated NPs of PLGA and validated in a MUC1-overexpressing breast cancer cells.

CD44 constitutes an additional appealing molecular candidate for anti-cancer targeted drug delivery [116]. One study has developed NPs coated with HA-appended PEG-PLGA polymers for the specific targeting of CD44-expressing triple-negative breast cancer cells. Such formulation, loaded with antisense miR-34a, depicted enhanced stability and potent in vivo toxicity [117]. A similar approach has been developed in the bladder cancer context, using CD44-targeting NPs loaded with anti-Bcl2 siRNAs [118]. Furthermore, PLGA-based NPs decorated with a Fab recognizing the CD44v6 variant highly expressed by gastrointestinal cancer cells have been developed [119].

Some other studies have employed methods to evaluate CEA, a cell surface glycoprotein overexpressed in several tumors, as a therapeutic target. A strategy was created for the targeting of triple-negative breast cancer using CEA-directed iron NPs, which depicted an enhanced tumor targeting capacity [120]. Additionally, CEA-targeting PLGA-PEG NPs loaded with paclitaxel have been demonstrated to selectively bind and trigger the death of CEA-expressing colorectal cancer cells [121].

### 4.3. Glycan-Targeting Nanoparticles

Targeting cancer-associated glycan epitopes is another promising strategy to achieve targeted drug delivery in cancer tissues. A commonly targeted glycan is SLe^a^, due to its high expression during malignant transformation. In this context, functionalized PLGA NPs with a mAb targeting SLe^a^ and loaded with paclitaxel and 5-FU have been developed [122]. More recently, Palma-Chavez et al. developed and characterized a PLGA-based multistage delivery system for improved drug delivery to SLe^a^-expressing inflamed vascular endothelium [123].

### 4.4. Nanoparticles Strategies Using Glycan-Binding Proteins

GBPs regulate the crosstalk between distinct sets of immune cell populations and tumor cells. By actively supporting the establishment of an immunosuppressive microenvironment, GBPs have emerged as promising targets of more efficient immunotherapies. Several studies have developed NPs functionalized with a mAb targeting Siglec-3 (CD33), which is abundantly expressed on the surface of acute myeloid leukaemia cells [124,125,126,127]. Furthermore, novel bi-specific liposomal-based NP T cell engagers (nanoTCEs) are conjugated with two mAbs targeting two distinct epitopes, one being a cancer antigen, and the other the Siglec-3 [124]. This formulation has shown to be efficient in the targeting and killing of AML cells, both in vitro and in vivo.

Another attractive target is Siglec-1 (CD169/sialoadhesin), due to is ubiquitous expression on macrophages. Chen et al. described a strategy using liposomal NPs with high binding affinity towards glycan-based ligands of Siglec-1 [128].

Several studies have been conducted using NPs functionalized with carbohydrate-recognizing lectins [129,130,131,132,133]. For instance, the mannose-binding concanavalin A was used to functionalize NPs and target sialic acids [129,133]. In addition, the conjugation of the wheat germ agglutinin to NPs loaded with anti-tumor chemotherapeutics has been shown to significantly enhance cancer cell-specific uptake, while minimizing off-target cytotoxicity [130]. Similar strategies have been employed for the targeting of cancer cells enriched in fucosylated antigens, such as Lewis x (Le^x^), using the fucose-recognizing lectins aleuria aurantia lectin [131] and the lotus tetragonolobus lectin [132].

## 5. Future Perspectives

Development of therapeutic strategies using NPs directed at glycans constitutes an emerging and promising field. Recent studies demonstrate that NPs, with their unique properties, represent excellent platforms to be developed and applied in the new era of personalized target therapy. They not only improve the release and therapeutic efficacy of anti-cancer drugs at the target side, but also reduce cytotoxicity and a drug’s off-target effects.

Several issues regarding NP formulation should be ensured in the future. The high level of consistency in the large-scale production, characterization, and reproducibility of the engineered NPs must be guaranteed. Besides, a better understanding of the NP interaction with the immune system and their in vivo biodistribution is crucial which, together with their evaluation in preclinical studies using suitable animal models, will set the basis for future preliminary human trials. In addition, the NPs’ fate and their biodegradability must be carefully evaluated, considering nanotoxicology issues.

The aberrant glycosylation landscape of tumor cells constitutes a major opportunity for developing advanced targeted therapies. The high prevalence of such glycosylation alterations expressed within the glycocalyx of tumor cells make them appealing target candidates for the antibody-based target delivery of NPs. Additionally, glycan recognizing molecules, such as antibodies, lectins, and other GBPs, unlock several potential strategies for clinical beneficial targeted therapeutics, ultimately improving patient clinical outcome.

Finally, the presently available nanoformulations targeting glycans have the potential to undergo improvements in order to ensure that these nanosystems could be efficiently translated into clinical practice.

## Figures and Tables

**Table 1:** Panel of antibody-drug conjugates (ADCs) approved by the Food and Drug Administration (FDA) and the European Medicines Agency (EMA).

ADC Formulation	Target Antigen	Antibody	Approved Clinical Application ^1^	Year of Approval	Approving Regulatory Entity
Brentuximab-vedotin (SGN-35, Adcetris)	CD30	Chimeric IgG1	Hodgkin’s lymphoma, ALCL, PTCL, MF	2011/2012	FDA and EMA
Trastuzumab-emtansine (T-DM1, Kadcyla)	ErbB2	Humanized IgG1	ErbB2-positive metastatic breast cancer	2013	FDA and EMA
Inotuzumab-ozogamicin (Besponsa)	CD22	Recombinant humanized IgG4	B cell precursor ALL	2017	FDA and EMA
Gemtuzumab-ozogamicin (Mylotarg) ^2^	CD33	Humanized IgG4	CD33-positive AML	2017/2018	FDA and EMA
Polatuzumab-vedotin (Polivy)	CD79	Humanized IgG1	DLBCL	2019/2020	FDA and EMA
Enfortumab-vedotin (ASG-22ME, Padcev)	Nectin-4	Human IgG1	Advanced urothelial cancer	2019	FDA
Trastuzumab-deruxtecan (DS-8201a, Enhertu)	ErbB2	Humanized IgG1	Metastatic ErbB2-positive breast cancer	2019	FDA
Sacituzumab-govitecan (IMMU-132, Trodelvy)	TROP2	Humanized IgG1	Triple-negative breast cancer	2020	FDA
Belantamab mafodotin (GSK2857916, Blenrep)	BCMA	Humanized IgG1	Relapsed or refractory multiple myeloma	2020 (orphan drug since 2017 by the EMA)	FDA

^1^ ALCL, anaplastic large cell lymphoma; PTCL, peripheral T cell lymphoma; MF, mycosis fungoides; ALL, acute lymphoblastic leukaemia; AML, acute myeloid leukaemia; DLBCL, diffuse large B cell lymphoma. ^2^ Gemtuzumab-ozogamicin was approved in 2000 and withdrawn from the market in 2010, then was re-approved in 2017 by the FDA.
